# 2091: Evaluating the validity and utility of surrogate endpoints in clinical trials of chronic kidney disease (CKD)

**DOI:** 10.1017/cts.2017.92

**Published:** 2018-05-10

**Authors:** Jian Ying, Andrew Redd, Tom Greene

**Affiliations:** The University of Utah School of Medicine, Salt Lake City, UT, USA

## Abstract

OBJECTIVES/SPECIFIC AIMS: The objective of this research is to determine under what conditions endpoints based on estimated glomerular filtration rate (eGFR) slope or on relatively small declines in eGFR provide valid and useful surrogate endpoints for pivotal clinical trials in chronic kidney disease (CKD) patients. METHODS/STUDY POPULATION: We consider 2 classes of surrogate endpoints. The first class includes endpoints defined by the average rate of change in eGFR during defined portions of the follow-up period of the trial, following initiation of the randomized treatment interventions. The second class includes composite endpoints defined by the time from randomization until the occurrence of a designated decline in eGFR or kidney failure. The true clinical endpoint is considered to be the time from randomization until kidney failure, irrespective of the trajectory in eGFR measurements prior to kidney failure. We apply statistical simulation to determine conditions under which alternative endpoints within the 2 classes are (1) valid surrogate endpoints, in the sense of preserving a low probability of rejecting the null hypothesis of no treatment effect on the surrogate endpoint when there is no treatment effect on the clinical endpoints and are also (2) useful surrogate endpoints, in the sense of providing increased statistical power that allows significant reductions in sample size and/or duration of follow-up. Input parameters for the simulations include (a) characteristics of the joint distribution of the longitudinal eGFR measurements and the time to occurrence of renal failure, (b) characteristics of the short-term and long-term effects of the treatment, and (c) design parameters, including the duration of accrual and follow-up and the spacing of eGFR measurements during the follow-up period. We use joint analyses of 19 treatment comparisons across 13 previous clinical trials of CKD patients to guide the selection of input parameters for the simulations. We apply longitudinal mixed effects models for analysis of endpoints based on eGFR slope, and Cox regression for analyses of the composite time-to-event endpoints. RESULTS/ANTICIPATED RESULTS: We have previously shown that surrogate endpoints defined by eGFR declines of 30% or 40% can provide valid and useful alternative endpoints in CKD clinical trials for interventions that do not produce short-term effects on eGFR which differ from the longer-term effects of the interventions. Other factors influencing the validity and utility of these endpoints include the average baseline eGFR, the mean rate of change in eGFR, and the extent to which the size of the treatment effect depends on the patient’s underling rate of eGFR decline. We will extend these results by presenting preliminary results describing conditions under which outcomes based on eGFR slope provide valid and useful alternatives to the clinical endpoint of time until occurrence of kidney failure. DISCUSSION/SIGNIFICANCE OF IMPACT: The statistical simulation strategy described in this research can be used during the design of clinical trials of chronic kidney disease to assist in the selection of endpoints that maximize savings in sample size and duration of follow-up while retaining a low risk of producing a false positive conclusion in the absence of a true effect of the treatment on the time until kidney failure.

